# Antibody–Drug Conjugates Plus Immune Checkpoint Inhibitors for Sustained Disease Control in Advanced Solid Tumors

**DOI:** 10.3390/cancers18213564

**Published:** 2026-11-06

**Authors:** Meiling Xu, Jun Yin, Yao Li, Liyuan Zhang

**Affiliations:** 1 Center of PRaG Therapy, The Second Affiliated Hospital of Soochow University, Suzhou 215004, China; 2 Center for Cancer Diagnosis and Treatment, The Second Affiliated Hospital of Soochow University, Suzhou 215025, China; 3 Laboratory for Combined Radiotherapy and Immunotherapy of Cancer, The Second Affiliated Hospital of Soochow University, Suzhou 215025, China; 4 Jiangsu Key Laboratory of Radiation Injury Prevention and Hazardous Substance Control, Suzhou 215123, China; 5 Jiangsu Nuclear Power Corporation, Lianyungang 222042, China

**Keywords:** antibody–drug conjugates, immune checkpoint inhibitors, advanced solid tumors, disease control, radiotherapy, treatment optimization

## Abstract

Antibody–drug conjugates (ADCs) combined with immune checkpoint inhibitors (ICIs) improve outcomes in selected advanced solid tumors, but clinical benefit does not establish pharmacological synergy. This narrative review evaluates disease control during ADC–ICI therapy and the implications for radiotherapy in recurrent or metastatic disease. Prolonged disease control is assessed primarily through progression-free survival in randomized trials; response duration provides complementary evidence among responders. Overall survival, quality of life, and toxicity determine the broader clinical value of treatment. Randomized trials support specific regimens in urothelial, breast, and lung cancers, whereas negative studies argue against generalization across drugs and diseases. Antigen expression, payload delivery, and the immune microenvironment may contribute to heterogeneous outcomes, but their clinical roles remain incompletely established. Reported treatment durations do not define optimal stopping times. Radiotherapy can address local symptoms, but whether it improves systemic disease control during ADC–ICI therapy remains unproven. We distinguish direct three-modality evidence from indirect ADC–radiotherapy and ICI–radiotherapy studies and identify priorities for patient selection, outcome assessment, and safety monitoring. Current evidence supports regimen-specific treatment selection and toxicity-directed modification, not routine radiotherapy addition or elective drug withdrawal across disease settings.

## Simple Summary

Antibody–drug conjugates deliver cancer-killing drugs to cells carrying a specific target. Combining them with immune checkpoint inhibitors can delay cancer progression and, in some settings, prolong survival. This review uses progression-free survival as the main measure of disease control, while considering response duration, quality of life, and side effects separately. Benefits depend on the drugs, cancer type, and previous treatment; they do not prove that the drugs act synergistically. For recurrent or metastatic cancer, an important question is whether radiotherapy can control a few progressing sites while drug treatment remains effective elsewhere. We distinguish this approach from symptom relief and from attempts to stimulate immunity against distant tumors. Evidence for adding radiotherapy to ADC–ICI treatment remains limited. Appropriate patient selection, tolerable treatment schedules, and comparative radiotherapy trials are needed before broader use.

## 1. Introduction

Antibody–drug conjugates (ADCs) deliver cytotoxic drugs to cells expressing a tumor-associated antigen and have expanded treatment options for advanced solid tumors. Responses can be substantial, but their duration varies; target loss and reduced payload sensitivity may lead to resistance [1,2,3]. Selected ADCs also promote antigen presentation and T-cell responses in experimental models, providing a rationale for combination with immune checkpoint inhibitors (ICIs) [4,5,6,7].

Randomized phase III trials have established clinical benefit in several settings. Enfortumab vedotin (EV) plus pembrolizumab and disitamab vedotin (DV) plus toripalimab improved progression-free survival (PFS) and overall survival (OS) more than platinum chemotherapy in advanced urothelial carcinoma [8,9]. Sacituzumab govitecan (SG) plus pembrolizumab improved PFS more than chemotherapy plus pembrolizumab in programmed death-ligand 1 (PD-L1)-positive triple-negative breast cancer (TNBC). Sacituzumab tirumotecan (sac-TMT) plus pembrolizumab improved PFS over pembrolizumab alone in PD-L1-positive non-small-cell lung cancer (NSCLC) [10,11]. However, other randomized trials found no significant improvement, indicating that benefit depends on the regimen and disease setting [12,13].

This narrative review focuses on adults with unresectable locally advanced, recurrent, or metastatic solid tumors and prioritizes peer-reviewed randomized trials, prospective clinical studies, and original mechanistic research. Conference-only reports were excluded irrespective of the direction of their findings; regulatory documents were used for dosing and safety information. Studies in localized disease are discussed only where they inform mechanisms, recurrence after previous therapy, or the feasibility of integrating radiotherapy. This is a selective critical synthesis, not a systematic review or meta-analysis.

We use prolonged disease control to mean a longer interval to progression or death, supported primarily by PFS in randomized trials. Duration of response (DoR) describes durability among responders; OS, quality of life, and toxicity establish the clinical value and acceptability of that control. No universal time threshold defines a durable response across tumor types. The relevance to radiotherapy–immunotherapy is a specific clinical question: when systemic ADC–ICI treatment controls most disease, can irradiation of selected lesions improve outcomes without compromising continued treatment? We therefore separate established local indications, experimental treatment of limited progression, and attempts to enhance systemic immunity. None can be inferred from the efficacy of the drug combination alone.

Throughout, clinical benefit refers to the observed comparison in a trial. Synergy would require evidence that the combination exceeds a prespecified expectation from the individual treatments; a favorable HR against chemotherapy or one constituent drug is insufficient. This distinction also applies to combinations incorporating radiotherapy.

## 2. Biological Rationale for ADC–ICI Therapy

### 2.1. Tumor Cell Killing and Antigen Presentation

The antibody, linker, and payload determine where an ADC acts and how tumor cells are killed. Antigen density, internalization, lysosomal processing, and drug efflux influence intracellular payload delivery. Linker stability and drug-to-antibody ratio affect both tumor exposure and systemic toxicity. Trastuzumab emtansine (T-DM1) uses a noncleavable linker and releases a poorly membrane-permeable DM1-containing catabolite, whereas cleavable-linker ADCs with membrane-permeable payloads can kill neighboring cells with lower target expression. This bystander effect may reduce the consequences of antigen heterogeneity, but it is not itself an immune response [1]. In trastuzumab deruxtecan (T-DXd) models, tumor- and stroma-derived cathepsin L can also release the deruxtecan (DXd) payload extracellularly [14]. This mechanism should not be assumed for other linker–payload combinations.

Selected ADCs induce signals associated with immunogenic cell death (ICD), including calreticulin exposure and release of adenosine triphosphate (ATP) and high-mobility group box 1 (HMGB1). Calreticulin can promote uptake of dying cells, while ATP and HMGB1 participate in recruitment and activation of antigen-presenting cells. Maytansine-bearing ADCs produced these signals in antigen-positive cells, and pyrrolobenzodiazepine- or tubulysin-containing ADCs showed immune-dependent antitumor effects in mouse models [15,16]. Dendritic cells must subsequently cross-present tumor antigens to CD8+ T cells; antigen release alone is insufficient [17].

ICD requires a functional, antigen-specific adaptive immune response, not simply the presence of these biomarkers. Consensus guidance cautions that damage-associated molecular signals are necessary in some models but insufficient to establish ICD; vaccination and rechallenge experiments in immunocompetent hosts provide stronger evidence [18]. Importantly, Garg et al. found that AY27 bladder cancer cells resisted the vaccination effect of established ICD-inducing treatments because defective calreticulin exposure impaired phagocytosis [19]. This is a context-dependent counterexample, not a failed replication of a particular ADC study. The available evidence therefore supports ICD as one possible mechanism for selected ADCs, without establishing its reproducibility across tumors or its causal role in patients.

Payload mechanisms may influence this sequence. Microtubule-disrupting agents arrest mitosis and can stimulate dendritic-cell maturation, while topoisomerase I inhibitors produce DNA damage that may engage innate immune signaling. T-DM1 increased T-cell infiltration in human epidermal growth factor receptor 2 (HER2)-positive models [4,5]. T-DXd increased dendritic-cell CD86 and tumor-cell major histocompatibility complex class I expression; additional experiments implicated Toll-like receptor 4 and stimulator of interferon genes (STING) in myeloid-cell activation and demonstrated Fc-dependent phagocytosis [6,14]. EV also induced ICD-associated signals and enhanced programmed cell death protein 1 (PD-1) blockade in preclinical models [20]. These findings are specific to the tested constructs, doses, and models, rather than evidence of a uniform immune effect of either payload class.

### 2.2. Sustaining T-Cell Responses with Checkpoint Blockade

ADC-induced inflammation may increase PD-L1 expression and restrain activated T cells. PD-1/PD-L1 blockade can release this inhibition when tumor antigens are presented and responsive T cells are present. T-DM1 increased sensitivity to combined cytotoxic T-lymphocyte-associated protein 4 (CTLA-4)/PD-1 blockade in HER2-positive mouse tumors, and T-DXd plus PD-1 blockade improved tumor control in an immunocompetent model [5,6]. Protection against tumor rechallenge and reduced efficacy after CD8+ T-cell depletion support immune involvement [16]. However, these experiments do not by themselves prove that ICD is the initiating mechanism or that a clinical combination exceeds the expected effects of its individual drugs.

**Figure 1:**
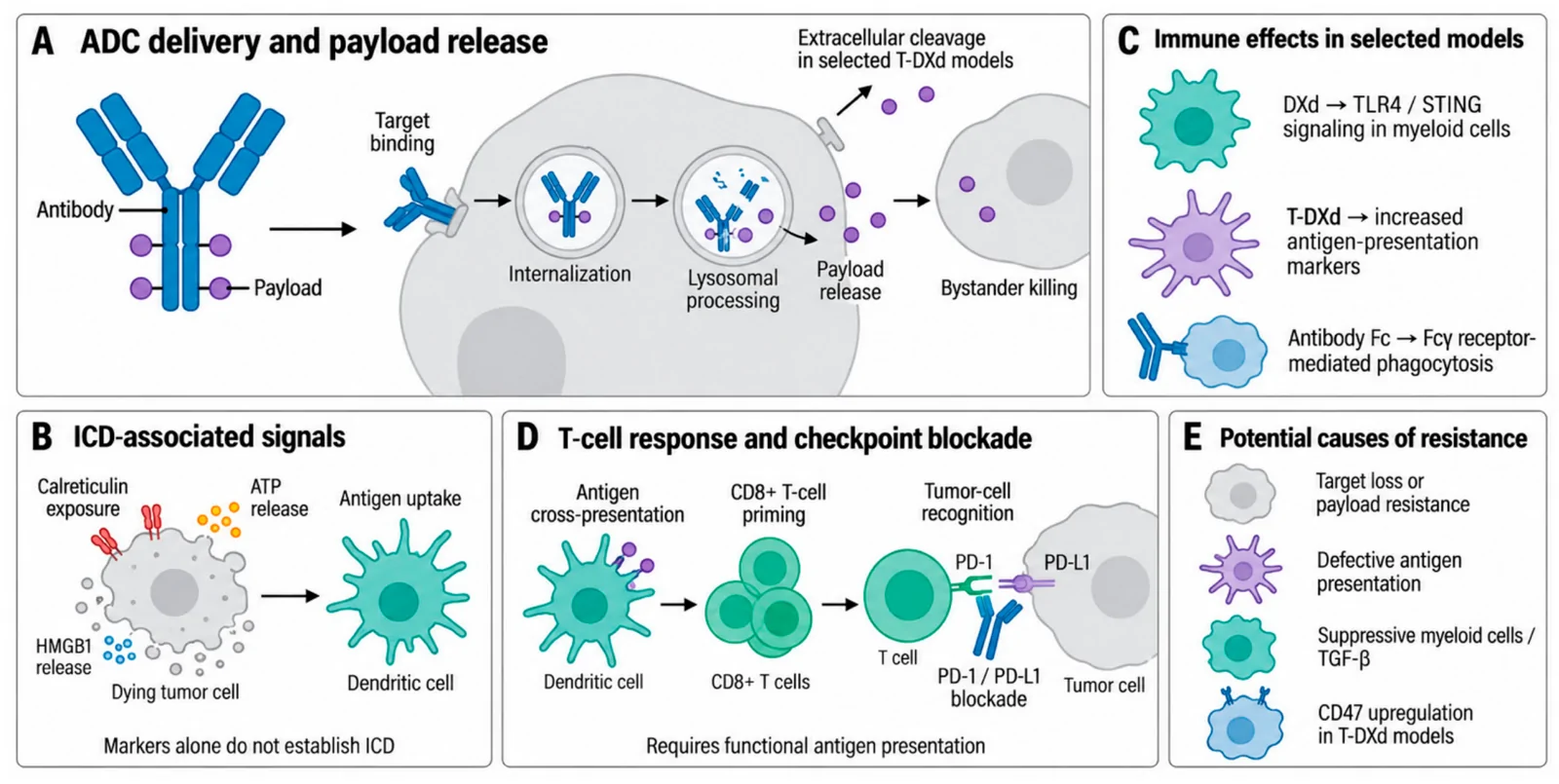
Proposed mechanisms and limitations of ADC–ICI therapy. (**A**) Target binding, internalization, and payload release; membrane-permeable payloads may kill neighboring cells. The blue Y-shaped structure represents the antibody and purple circles represent payload molecules. Connectors are schematic, not chemical structures. Extracellular cleavage is supported by selected T-DXd models [1,14]. (**B**) ICD-associated signals may facilitate antigen uptake, but their detection alone does not establish immunogenic cell death [15,16,18,19]. (**C**) Innate signaling, antigen-presentation changes, and Fc-dependent phagocytosis are observations from selected experimental models [4,5,6,14,20]. (**D**) Cross-presentation and T-cell recognition provide the rationale for checkpoint blockade; this diagram does not demonstrate clinical synergy. (**E**) Potential causes of resistance. CD47 upregulation is principally preclinical, whereas human leukocyte antigen (HLA) reduction, TGF-β-related changes, HER2 loss, and TOP1 alterations have been reported in patient specimens [2,3,14,21,22,23]. All arrows indicate proposed biological relationships, not strength of evidence. Neither tissue associations nor preclinical immune effects establish the mechanism of clinical benefit.

Defective antigen presentation, T-cell exclusion, suppressive myeloid cells, and other inhibitory pathways may prevent checkpoint blockade from sustaining an immune response. DXd-induced CD47 upregulation restricted phagocytosis in T-DXd models [14]. The antibody Fc region may recruit Fcγ receptor-bearing effector cells, but this depends on antibody properties and the local balance of activating and inhibitory receptors. Cytotoxic payloads may also injure proliferating immune cells. Thus, antigen release, immune-cell recruitment, and checkpoint inhibition are complementary requirements, not a sequence that necessarily occurs in every patient [7]. Figure 1 separates these proposed mechanisms from the evidence needed to validate them.

### 2.3. Evidence from Patient Specimens

Studies of patient specimens suggest that ADC treatment can alter the immune microenvironment, although most evaluated monotherapy. In a gastric cancer study of T-DXd, paired biopsies showed a tendency toward increased CD8+ T-cell infiltration, without a statistically significant increase [22]. DESTINY-Gastric06 analyses identified early changes in immune and antigen-presentation pathways, followed at resistance by increased transforming growth factor-β (TGF-β) signaling and PD-1 expression [23]. Matched gastric cancer specimens also linked resistance to reduced human leukocyte antigen expression [21]. These observations support clinical investigation of immune-mediated resistance but do not demonstrate that an ICI reverses it.

In DAISY, T-DXd produced responses in some HER2 immunohistochemistry (IHC) 0 tumors, although response rates were higher in HER2-overexpressing disease [24]. This finding does not establish extracellular cleavage or immune activation as the mechanism of response. Differences in antigen expression and immune-cell composition also limit translation from mouse models [25]. Serial patient samples are needed to distinguish immune changes from target loss or payload resistance and determine how these processes relate to response duration during combination treatment.

### 2.4. Why Outcomes Differ Across ADC–ICI Regimens

The outcomes in Table likely reflect both drug properties and the biological setting, but the trials were not designed to identify the molecular cause of success or failure. T-DM1 and cleavable-linker, membrane-permeable payload ADCs differ in intracellular processing and bystander killing [1]. Such differences could matter in heterogeneous tumors, yet they cannot by themselves explain the negative KATE2 result, particularly because T-DM1 showed immune effects preclinically [5,12]. Fc function, payload sensitivity, and immune-cell injury may also alter the balance between tumor killing and immune activation.

Tumor immune context is equally important. ASCENT-04 selected PD-L1-positive TNBC, whereas SACI-IO HR+ studied hormone receptor-positive disease without the same PD-L1 restriction [10,13]. Their contrasting results are compatible with differences in immune responsiveness, but do not prove that PD-L1 or breast cancer subtype alone explains benefit. Target loss, defective HLA expression, suppressive myeloid cells, and immune exclusion can each interrupt the proposed mechanism. Conversely, direct ADC cytotoxicity may improve PFS even when additional immune activation is limited.

Clinical design also affects the apparent contrast between positive and negative studies. EV-302 and RC48-C016 compared the combination with chemotherapy, whereas KATE2 and SACI-IO HR+ tested ICI addition to an ADC [8,9,12,13]. Different comparators, treatment lines, biomarker criteria, and follow-up periods preclude attributing their divergent results to payload chemistry alone. Molecular explanations should therefore guide prospective sampling and hypothesis testing, not retrospectively label a regimen as synergistic.

## 3. Clinical Evidence for Improved Outcomes

### 3.1. Urothelial Carcinoma

Urothelial carcinoma provides the strongest evidence that an ADC–ICI regimen can improve both disease control and survival. EV-302 enrolled 886 patients with untreated locally advanced or metastatic disease and compared EV plus pembrolizumab with gemcitabine plus cisplatin or carboplatin. Median PFS was 12.5 versus 6.3 months (hazard ratio [HR] 0.45, 95% confidence interval [CI] 0.38–0.54), and median OS was 31.5 versus 16.1 months (HR 0.47, 95% CI 0.38–0.58) [8]. The combination benefited both cisplatin-eligible and cisplatin-ineligible patients and did not require selection by Nectin-4 or PD-L1 expression [26].

RC48-C016 enrolled 484 previously untreated patients in China with HER2 IHC 1+, 2+, or 3+ urothelial carcinoma. DV plus toripalimab prolonged median PFS from 6.5 to 13.1 months (HR 0.36, 95% CI 0.28–0.46) and median OS from 16.9 to 31.5 months at the interim analysis (HR 0.54, 95% CI 0.41–0.73), after 18.2 months of median follow-up. Median response duration was 14.6 versus 5.6 months [9]. These results extend the encouraging findings of RC48-C014 to a randomized phase III comparison [27]. They support this regimen in the HER2-expressing population studied, without establishing superiority over EV plus pembrolizumab.

Neither phase III trial included an ADC monotherapy group. In randomized EV-103 cohort K, confirmed objective response rates (ORRs) were 64.5% with EV plus pembrolizumab and 45.2% with EV alone; 12-month PFS rates were 55.1% and 35.8% [28]. These findings are descriptive because no formal between-group efficacy comparison was planned.

Results after platinum treatment are less consistent. MORPHEUS-UC found no improvement in response, PFS, or OS with SG plus atezolizumab over atezolizumab alone, although the study was small [29]. JAVELIN Bladder Medley reported longer PFS with SG plus avelumab maintenance (HR 0.49, 95% CI 0.31–0.76). However, the analysis supplemented the randomized control group with propensity score-weighted JAVELIN Bladder 100 data, and grade ≥ 3 treatment-related adverse events (TRAEs) were more frequent with the combination [30]. Confirmation is needed before adopting this more intensive maintenance approach. Early-phase urothelial cancer cohorts are summarized in Table A1.

### 3.2. Breast Cancer

ASCENT-04 compared SG plus pembrolizumab with chemotherapy plus pembrolizumab as first-line treatment in 443 patients with advanced TNBC and PD-L1 combined positive score (CPS) ≥ 10. Median PFS was 11.2 versus 7.8 months (HR 0.65, 95% CI 0.51–0.84). ORRs were 60% and 53%, and median DoR was 16.5 and 9.2 months [10]. Thus, replacing chemotherapy with SG improved PFS and was associated with longer responses; OS remained immature. The trial did not compare the combination with SG alone.

By contrast, KATE2 found no significant PFS improvement when atezolizumab was added to T-DM1 in 202 patients with previously treated HER2-positive breast cancer. Median PFS was 8.2 versus 6.8 months (HR 0.82, 95% CI 0.55–1.23; *p* = 0.33), and serious adverse events occurred in 33% versus 19% [12]. SACI-IO HR+ likewise found no significant PFS improvement with SG plus pembrolizumab over SG alone in hormone receptor-positive, HER2-negative metastatic disease (8.4 versus 6.7 months; HR 0.76, 95% CI 0.48–1.19; *p* = 0.12) [13]. Exploratory PD-L1 subgroup findings do not justify routine use of these combinations.

In the nonrandomized BEGONIA cohorts, datopotamab deruxtecan (Dato-DXd) plus durvalumab produced ORRs of 79.0% in PD-L1-unselected untreated TNBC and 81.8% in PD-L1-high disease. Median DoR and PFS in the former cohort were 17.6 and 14.0 months [31]. These results warrant randomized evaluation. Additional early-phase breast cancer cohorts are summarized in Table A1.

### 3.3. Non-Small-Cell Lung Cancer

OptiTROP-Lung05 randomized 413 previously untreated patients at 68 centers in China with advanced NSCLC, PD-L1 tumor proportion score (TPS) ≥ 1%, and no targetable genomic alterations to sac-TMT plus pembrolizumab or pembrolizumab alone. At the prespecified interim analysis, after 10.5 months of median follow-up, median PFS was not reached versus 5.7 months (HR 0.35, 95% CI 0.26–0.47). ORRs were 70% and 42%; grade ≥ 3 treatment-emergent adverse events occurred in 55% and 31%, respectively [11]. Both the TPS 1–49% and TPS ≥ 50% subgroups favored the combination.

The PFS benefit in OptiTROP-Lung05 is established against pembrolizumab alone, not chemoimmunotherapy, which is an important alternative for chemotherapy-eligible patients with TPS 1–49%. Longer follow-up is needed to assess OS and cumulative toxicity; validation in other populations would clarify applicability beyond China.

More recently, OptiTROP-Lung06 evaluated a distinct population: 455 patients with untreated PD-L1 TPS < 1% nonsquamous NSCLC without targetable genomic alterations. Sac-TMT plus pembrolizumab improved median PFS over platinum–pemetrexed plus pembrolizumab (15.0 versus 5.7 months; HR 0.49, 95% CI 0.36–0.65) at a prespecified interim analysis with 9.7 months of median follow-up. ORRs were 50.2% and 47.3%, and median DoR was 13.6 and 6.8 months; OS remained immature [32]. This comparison supports replacing chemotherapy with the ADC in the studied regimen, not the efficacy of adding pembrolizumab to sac-TMT alone.

The phase Ib TROPION-Lung02 study evaluated the trophoblast cell-surface antigen 2 (TROP2)-directed ADC Dato-DXd plus pembrolizumab, with or without platinum chemotherapy. In treatment-naive patients, ORR, median DoR, and median PFS were 54.8%, 20.1 months, and 11.2 months with the doublet (*n* = 42), compared with 55.6%, 13.7 months, and 6.8 months with the triplet (*n* = 54). Grade ≥ 3 TRAEs occurred in 37.1% and 59.7% of the full safety cohorts (*n* = 70 and 72), which also included previously treated patients [33]. The nonrandomized comparison cannot establish superiority of the doublet or determine whether adding platinum improves outcomes.

In EVOKE-02, SG plus pembrolizumab produced median PFS of 13.1 months in the TPS ≥ 50% cohort and 7.0 months in the TPS < 50% cohort [34]. These uncontrolled findings require randomized confirmation. Table 1 summarizes selected prospective studies by tumor type and separates randomized from nonrandomized evidence. Table A1 retains early-phase cohorts that inform response durability, biomarker selection, or toxicity, including studies without convincing efficacy; they are not additional proof of comparative benefit. Extended EV-302 follow-up is considered separately in Section 4.2 [35].

### 3.4. Gastrointestinal and Gynecologic Cancers

Evidence in other solid tumors remains predominantly nonrandomized. A 57-patient phase II study in gastric or gastroesophageal junction adenocarcinoma with HER2 IHC 2+ or 3+, irrespective of in situ hybridization status, reported a confirmed ORR of 89.5% with DV, tislelizumab, and S-1 [36]. Without a control group, this result does not establish an improvement over standard first-line therapy.

In innovaTV 205, responses to tisotumab vedotin plus pembrolizumab remained ongoing in 6 of 13 first-line and 5 of 12 later-line responders after more than five years of follow-up. Median PFS was nevertheless 5.3 and 5.6 months in the respective cervical cancer cohorts. In the first-line arm H, which also included carboplatin with or without bevacizumab, ORR was 65.8%, median PFS was 10.6 months, and grade ≥ 3 AEs related to any study treatment occurred in 86.8% of patients [37]. These nonrandomized cohorts differed in treatment and follow-up; their results do not establish the relative benefit of adding chemotherapy or bevacizumab.

In platinum-resistant ovarian cancer, mirvetuximab soravtansine plus pembrolizumab produced an ORR of 31% and median PFS of 4.2 months. Pneumonitis occurred in 25% of treated patients, including grade ≥ 3 events in 4% [38]. These findings do not establish an improvement over ADC monotherapy. Across these gastrointestinal and gynecologic cohorts, single-arm response estimates should not be interpreted as evidence that adding an ICI improves outcomes.

A recent single-arm phase II study also cautions against extrapolation across HER2-expressing tumors. Among 15 patients with microsatellite-stable/proficient-mismatch-repair, HER2-positive metastatic colorectal cancer after two previous systemic treatment lines, DV plus pembrolizumab produced two partial responses (ORR 13.3%, 95% CI 1.7–40.5%) and median PFS of 2.9 months [39]. These limited results do not support transferring the urothelial cancer findings to colorectal cancer solely on the basis of HER2 expression.

**Table 1:** Selected prospective studies of ADC–ICI therapy, organized by tumor type and study design.

Trial and Design	Population	Treatment Groups (n)	PFS	OS	Tumor Response	Selected Safety Findings
**A. Urothelial carcinoma**						
EV-302 [8,26] Phase III; open-label; 1:1; global Median FU 17.2 mo	Untreated la/mUC; eligible for cisplatin or carboplatin	EV + pembrolizumab (442) vs. gemcitabine + cisplatin or carboplatin (444) ^a^	BICR: 12.5 (10.4–16.6) vs. 6.3 (6.2–6.5) HR 0.45 (0.38–0.54); *p* < 0.001	31.5 (25.4–NE) vs. 16.1 (13.9–18.3) HR 0.47 (0.38–0.58); *p* < 0.001	ORR 67.7% (63.1–72.1) vs. 44.4% (39.7–49.2); *p* < 0.0001	Grade ≥ 3 TRAEs 55.9 vs. 69.5 Serious TEAEs 50 vs. 39 TEAEs leading to discontinuation of any study drug 40 vs. 21
**RC48-C016** [9] Phase III; open-label; 1:1; China Median FU 18.2 mo	Untreated HER2-expressing (IHC 1+ to 3+) la/mUC	DV + toripalimab (243) vs. gemcitabine + cisplatin or carboplatin (241)	BICR: 13.1 (11.1–16.7) vs. 6.5 (5.7–7.4) HR 0.36 (0.28–0.46); *p* < 0.001	Interim: 31.5 (21.7–NR) vs. 16.9 (14.6–21.7) HR 0.54 (0.41–0.73); *p* < 0.001	ORR 76.1% (70.3–81.3) vs. 50.2% (43.7–56.7) Median DoR 14.6 (11.3–18.7) vs. 5.6 (5.3–5.8)	Grade ≥ 3 TRAEs 55.1 vs. 86.9
EV-103 cohort K [28] Randomized phase Ib/II; open-label; 1:1; no formal comparison Median FU 14.8 vs. 15.0 mo	Cisplatin-ineligible, untreated la/mUC	EV + pembrolizumab (76 treated) vs. EV (73 treated); 151 randomized	BICR 12-month rate: 55.1 vs. 35.8%	12-month rate: 80.7 vs. 70.7% Median 22.3 (19.1–NR) with the combination	BICR, confirmed ORR 64.5% (52.7–75.1) vs. 45.2% (33.5–57.3) CR 10.5% with the combination	Combination: grade ≥ 3 skin reactions 21.1; treatment-related deaths 3.9
MORPHEUS-UC [29] Randomized phase Ib/II umbrella; open-label; not powered for comparison Median FU 13.2 vs. 17.8 mo	Platinum-treated la/mUC; ICI-naive	Atezolizumab + SG (15 randomized/treated) vs. atezolizumab (30 randomized; 29 treated/analyzed)	Investigator: 3.5 (2.1–8.3) vs. 6.1 (3.5–10.2)	14.6 (8.3–NE) vs. 18.8 (10.6–31.5)	Confirmed ORR 6.7% (0.2–32.0) vs. 27.6% (12.7–47.2)	Grade 3–4 AEs 73.3 vs. 31.0 Discontinuation due to AEs 20.0 vs. 0
JAVELIN Bladder Medley [30] Randomized phase II platform; open-labelInterim cutoff: 16 Sep 2024	la/mUC without progression after first-line platinum-based chemotherapy (maintenance)	Avelumab + SG (74) vs. avelumab (37) ^c^	Investigator: 11.17 (7.43–NE) vs. 3.75 (3.32–6.77) HR 0.49 (0.31–0.76) Prespecified efficacy boundary: HR ≤ 0.60	Interim: NR vs. 23.75; HR 0.79 (0.42–1.50)	ORR 24.3% (15.1–35.7) vs. 2.7% (0.1–14.2) ^c^	Grade ≥ 3 TRAEs 69.9 vs. 0
**B. Breast cancer**						
**ASCENT-04** [10] Phase III; open-label; 1:1; international Median FU 14.0 mo	Untreated la/m TNBC; PD-L1 CPS ≥ 10; recurrence ≥ 6 mo after curative-setting therapy	SG + pembrolizumab (221) vs. chemotherapy + pembrolizumab (222)	BICR: 11.2 (9.3–16.7) vs. 7.8 (7.3–9.3) HR 0.65 (0.51–0.84); two-sided *p* < 0.001	Immature	ORR 60% (53–66) vs. 53% (46–60) Median DoR 16.5 (12.7–19.5) vs. 9.2 (7.6–11.3)	Grade ≥ 3 AEs 71 vs. 70 AEs leading to discontinuation of ≥1 study drug 12 vs. 31 AEs leading to death 3 vs. 3
**KATE2** [12] Randomized phase II; double-blind; 2:1; unmasked early for futility Median FU 8.5 vs. 8.4 mo	HER2-positive advanced BC after trastuzumab and a taxane	T-DM1 + atezolizumab (133) vs. T-DM1 + placebo (69)	Investigator: 8.2 (5.8–10.7) vs. 6.8 (4.0–11.1) HR 0.82 (0.55–1.23); *p* = 0.33	Immature at primary analysis	—	Serious AEs 33 vs. 19 Grade ≥ 3 thrombocytopenia 13 vs. 4 One treatment-related death (hemophagocytic syndrome)
**SACI-IO HR+** [13] Randomized phase II; 1:1 Median FU 15.5 mo	Hormone receptor-positive, HER2-negative MBC after endocrine therapy and 0–1 lines of chemotherapy; no prior TOP1 inhibitor	SG + pembrolizumab vs. SG (104 treated in total)	8.4 vs. 6.7 HR 0.76 (0.48–1.19); *p* = 0.12	20.0 vs. 18.0; *p* = 0.18	ORR 28.8% vs. 19.2%; *p* = 0.36	Most frequent grade ≥ 2 AEs: neutropenia, alopecia, fatigue, anemia
**C. Non-small-cell lung cancer**						
**OptiTROP-Lung05** [11] Phase III; open-label; 1:1; China (68 centers) Median FU 10.5 mo (interim)	Untreated advanced NSCLC; PD-L1 TPS ≥ 1%; no targetable genomic alterations	sac-TMT + pembrolizumab (208) vs. pembrolizumab (205)	BICR: NR (13.6–NE) vs. 5.7 (4.3–7.0) HR 0.35 (0.26–0.47); *p* < 0.0001	Immature	ORR 70% (63–76) vs. 42% (35–49) ^b^	Grade ≥ 3 TEAEs 55 vs. 31
OptiTROP-Lung06 [32] Phase III; open-label; 1:1 Median FU 9.7 mo (interim)	Untreated la/m nonsquamous NSCLC; PD-L1 TPS < 1%; no targetable genomic alterations	sac-TMT + pembrolizumab (229) vs. platinum–pemetrexed + pembrolizumab (226)	BICR: 15.0 vs. 5.7 HR 0.49 (0.36–0.65); *p* < 0.001	Immature; HR 0.58 (0.38–0.88)	ORR 50.2% vs. 47.3% Median DoR 13.6 vs. 6.8	Grade ≥ 3 treatment-emergent AEs 47.6 vs. 60.6
**D. Gastric and gastroesophageal junction cancer—nonrandomized**						
Li et al. [36] Single-arm phase II	First-line G/GEJ adenocarcinoma; HER2 IHC 2+ or 3+, irrespective of ISH status	DV 2.5 mg/kg + tislelizumab 200 mg q3w + S-1 on days 1–14 (n = 57)	13.8 (10.3–24.0)	31.9 (22.1–NR)	ORR 89.5% (78.5–96.0) Median DoR 13.3 (9.6–NR)	Grade ≥ 3 TRAEs 64.9
**E. Gynecologic cancers—nonrandomized**						
innovaTV 205 arm E [37] Phase Ib/II	First-line recurrent or metastatic cervical cancer	Tisotumab vedotin 2 mg/kg + pembrolizumab 200 mg q3w (n = 33)	5.3 (4.0–12.2)	30.7 (13.0–NR)	ORR 40.6% (23.7–59.4) Median DoR NR (3.0–NR)	Grade ≥ 3 AEs related to either study drug: 45.5
innovaTV 205 arm F [37] Phase Ib/II	Second- or third-line recurrent or metastatic cervical cancer	Tisotumab vedotin 2 mg/kg + pembrolizumab 200 mg q3w (n = 35)	5.6 (2.7–14.2)	15.3 (9.9–35.3)	ORR 35.3% (19.7–53.5) Median DoR 18.2 (4.2–NR)	Grade ≥ 3 AEs related to either study drug: 48.6
innovaTV 205 arm H [37] Phase Ib/II	First-line recurrent or metastatic cervical cancer	Tisotumab vedotin + pembrolizumab + carboplatin, with or without bevacizumab (n = 38)	10.6 (6.8–17.8)	28.0 (19.7–NR)	ORR 65.8% (48.6–80.4) Median DoR 13.3 (6.1–NR)	Grade ≥ 3 AEs related to any study treatment: 86.8
Matulonis et al. [38] Phase Ib	Platinum-resistant ovarian cancer; study-defined FRα expression	Mirvetuximab soravtansine 6 mg/kg AIBW + pembrolizumab 200 mg q3w (n = 56)	4.2 (2.8–5.6)	—	ORR 31% (19–45) Median DoR 8.0 (4.2–NR)	Pneumonitis: any grade 25; grade ≥ 3, 4%
**F. Colorectal cancer—nonrandomized**						
Xu et al. [39] Single-arm phase II	MSS/pMMR, HER2-positive mCRC after two prior systemic lines	DV 2.5 mg/kg q2w + pembrolizumab 400 mg q6w (15 enrolled; 14 treated)	2.9 (2.3–NR)	NR (16.7–NR)	ORR 13.3% (1.7–40.5) DoR —	Grade 3–4 neutropenia 14.3% (2/14 treated patients)

Sections A–C: randomized studies; results are experimental/control. Sections D–F: nonrandomized cohorts. Times are months; CIs are 95%. n is randomized in A–C unless indicated and treated in D–F. Safety n: EV-302, 440/433; RC48-C016, 243/222; ASCENT-04, 221/220; OptiTROP-Lung05, 208/204; JAVELIN, 73/36; MORPHEUS-UC, 15/29; KATE2, 132/68. EV-302 ORR was assessed in patients with measurable disease at baseline (437/441). Safety rates are percentages; event definitions and grades are study-specific. DoR comparisons include responders only and are descriptive. EV-302: primary analysis; extended follow-up is discussed in Section 4.2 [35]. ^a^ Avelumab maintenance: 131/444 control patients (30%) [26]. ^b^ ORR CIs not adjusted for multiplicity [11]. ^c^ JAVELIN PFS/OS analyses used propensity score-weighted external controls; ORR used randomized patients and the post-platinum baseline [30]. —, not tabulated. ADC, antibody–drug conjugate; AE, adverse event; BC, breast cancer; BICR, blinded independent central review; CI, confidence interval; CPS, combined positive score; CR, complete response; DoR, duration of response; DV, disitamab vedotin; EV, enfortumab vedotin; FU, follow-up; HER2, human epidermal growth factor receptor 2; HR, hazard ratio; ICI, immune checkpoint inhibitor; IHC, immunohistochemistry; la/m, locally advanced or metastatic; MBC, metastatic breast cancer; NE, not estimable; NR, not reached; NSCLC, non-small-cell lung cancer; ORR, objective response rate; OS, overall survival; PD-L1, programmed death-ligand 1; PFS, progression-free survival; sac-TMT, sacituzumab tirumotecan; SG, sacituzumab govitecan; T-DM1, trastuzumab emtansine; TEAE, treatment-emergent adverse event; TNBC, triple-negative breast cancer; TOP1, topoisomerase I; TPS, tumor proportion score; TRAE, treatment-related adverse event; UC, urothelial carcinoma. OptiTROP-Lung06 values are from the peer-reviewed interim report; OS is immature [32]. Section F: efficacy n = 15 enrolled; safety n = 14 treated because one patient withdrew before treatment [39]. MSS, microsatellite stable; pMMR, proficient mismatch repair; mCRC, metastatic colorectal cancer; q6w, every 6 weeks. Sections D–E: In innovaTV 205, efficacy populations were 32/34/38 and safety populations were 33/35/38 in arms E/F/H, respectively. Arm H used tisotumab vedotin 2 mg/kg, pembrolizumab 200 mg, and carboplatin AUC 5, with or without bevacizumab 15 mg/kg, q3w; its follow-up was shorter than that of arms E/F. S-1 was administered at 40–60 mg twice daily on days 1–14 of each 21-day cycle. The ovarian cancer study included 55 response-evaluable patients; FRα eligibility required ≥2+ staining in ≥25% of cells during dose escalation and ≥50% during expansion. Its safety column reports pneumonitis specifically, not all high-grade AEs. Li et al. and innovaTV 205 arm H included additional chemotherapy (and optional bevacizumab in arm H), whereas arms E/F and the ovarian cohort evaluated ADC–ICI doublets. None had a randomized comparator; cross-study differences do not establish relative efficacy. AIBW, adjusted ideal body weight; FRα, folate receptor alpha; G/GEJ, gastric/gastroesophageal junction; ISH, in situ hybridization; q3w, every 3 weeks; other abbreviations are defined above.

## 4. Clinical Significance of Prolonged Disease Control

### 4.1. Response Duration and Progression-Free Survival

PFS in randomized trials is the principal measure of prolonged disease control used in this review: it measures time from randomization to progression or death and includes patients regardless of response. Median PFS, the HR, follow-up duration, and, when reported, landmark PFS rates should be interpreted together. DoR measures time from response to progression or death among responders and is complementary, not interchangeable. Between-group DoR comparisons are descriptive because the responding subsets no longer retain the comparability provided by randomization. Neither a PFS advantage nor a response persisting during treatment demonstrates treatment-free control or immune memory.

Concordant PFS and DoR findings in ASCENT-04 support benefit beyond initial tumor shrinkage [10]. In contrast, long-lasting responses in some innovaTV 205 patients coexisted with relatively short median PFS [37]. A high ORR or a few long-term responders is therefore insufficient to establish sustained benefit across the treated population.

### 4.2. Overall Survival and Treatment After Progression

Extended follow-up provides stronger evidence of sustained benefit. At approximately 2.5 years in EV-302, median OS was 33.8 versus 15.9 months (HR 0.51, 95% CI 0.43–0.61), and median DoR was 23.3 versus 7.0 months [35]. The nonrandomized, 45-patient EV-103 dose-escalation/cohort A reported estimated five-year OS of 41.5% (95% CI 26.5–56.0) [40], documenting long-term survivors without providing a comparative estimate. Where OS remains immature, a PFS advantage should not be presented as a proven survival benefit.

Survival also reflects subsequent treatment. In EV-302, 30% of chemotherapy-assigned patients received avelumab maintenance and 12% subsequently received EV [26]; ASCENT-04 allowed subsequent SG [10]. These pathways did not guarantee access to both drug classes. Whether upfront combination therapy outperforms a planned sequence therefore remains unresolved.

### 4.3. Treatment Burden and Quality of Life

Similar high-grade adverse-event rates do not necessarily imply a similar treatment burden. In ASCENT-04, grade ≥ 3 events occurred in 71% and 70%, but adverse events led to discontinuation of at least one study drug in 12% and 31% [10]. Persistent lower-grade neuropathy, stomatitis, fatigue, or ocular symptoms can also impair daily life [41]. Drug-specific discontinuation and patient-reported symptoms are therefore important complements to aggregate safety rates.

Among 731 EV-302 patients with baseline questionnaires, a descriptive analysis showed a least-squares mean between-group difference of 2.54 points in global health status/quality-of-life change through week 26 (95% CI 0.41–4.67), favoring EV plus pembrolizumab; time to pain progression did not differ significantly [42]. This small difference is consistent with preservation of overall quality of life, not a large improvement. Missing later assessments limit conclusions about prolonged treatment.

### 4.4. Implications for Clinical Practice

The evidence supports specific ADC–ICI regimens, not routine ICI addition to every effective ADC. Randomized PFS improvements show that the tested strategy delays progression; mature OS and patient-reported outcomes determine whether this translates into broader benefit. The magnitude of PFS improvement does not establish pharmacological synergy, and negative trials caution against selecting combinations from mechanism alone [12,13,29]. For recurrent or metastatic disease, treatment should match the studied population and account for previous systemic therapy and residual toxicity. Elective drug withdrawal and radiotherapy intended to extend systemic control require their own comparative evidence. Table 2 summarizes the resulting treatment considerations without ranking regimens across trials.

## 5. Selecting Patients for ADC–ICI Therapy

### 5.1. Disease Setting and Previous Treatment

Patient selection should start with whether disease is an unresectable local recurrence, de novo metastatic disease, or metastatic recurrence after curative-intent treatment. These populations may differ in previous ADC or ICI use, treatment-free interval, disease burden, and residual toxicity. First-line treatment for advanced disease does not necessarily mean no previous systemic treatment. Trial eligibility and subgroup data should be checked before applying a positive overall result to early relapse or established ICI resistance. Progression during an ICI and recurrence after a long treatment-free interval should not be treated as equivalent clinical situations.

ASCENT-04 permitted previous perioperative therapy, including PD-1/PD-L1 inhibition, if recurrence occurred after a protocol-defined interval of at least six months [10]. This does not establish efficacy in primary ICI-resistant disease. Perioperative EV plus pembrolizumab has improved event-free survival in muscle-invasive bladder cancer, making recurrence after both drug classes increasingly relevant [43,44]. For such patients, the previous ADC target and payload, residual toxicity, and disease-specific alternatives should guide treatment; results from untreated metastatic disease may not apply.

For an isolated locoregional recurrence, assess whether surgery or definitive salvage radiotherapy remains appropriate before applying a palliative metastatic treatment strategy. For metastatic recurrence, record previous radiation fields, prior ICI response, and residual neuropathy or lung injury. The currently reported ADC–ICI trials do not establish equivalent benefit in de novo metastatic disease and every post-treatment recurrence subgroup. Prior radiotherapy is also relevant to the feasibility and toxicity of reirradiation, rather than a validated predictor of response to ADC–ICI therapy.

### 5.2. Biomarkers for Treatment Selection

Biomarker testing should follow regimen-specific evidence. RC48-C016 required HER2 IHC 1+ to 3+, whereas EV-302 did not require Nectin-4 testing [8,9]. ASCENT-04 required PD-L1 CPS ≥ 10 and OptiTROP-Lung05 required TPS ≥ 1% [10,11]. The separate OptiTROP-Lung06 trial supports a different setting, PD-L1 TPS < 1% nonsquamous NSCLC, against chemoimmunotherapy [32]. These results do not make PD-L1 irrelevant: histology, comparator, and regimen remain essential. CPS and TPS are not interchangeable, and no single biomarker selects patients for all ADC–ICI combinations.

Markers associated with ADC response do not necessarily identify patients who benefit more from combination treatment. NECTIN4 amplification was associated with EV monotherapy response [45]. High pretreatment NECTIN4 RNA expression was also associated with response and OS in 322 patients receiving EV plus pembrolizumab, but not in a separate pembrolizumab-treated cohort [46]. These nonrandomized findings do not establish predictive value. Low NECTIN4 expression should not currently exclude patients from a regimen supported by a biomarker-unselected randomized trial.

Conventional TROP2 IHC has not consistently predicted outcomes with SG [13,34,47]. Computational measures of TROP2 distribution showed exploratory associations in TROPION-Lung02, but confidence intervals for the PFS and OS hazard ratios included 1 [33]. Spatial target-expression features were associated with response to a HER2-directed ADC in early breast cancer [48]. Neither approach is validated for selecting advanced-disease combination therapy. Likewise, ADC monotherapy efficacy in ICI-ineligible TNBC does not justify use of the combination in that population [49,50].

In a retrospective series of 110 patients receiving EV with or without pembrolizumab, persistent circulating tumor DNA (ctDNA) positivity within 12 weeks was associated with shorter PFS and OS [51]. This is prognostic evidence from a heterogeneous cohort, not a validated test for choosing combination therapy. A change in ctDNA alone should not prompt withdrawal or replacement of clinically effective treatment without prospective evidence that such a decision improves outcomes.

### 5.3. Fitness, Comorbidity, and Treatment Feasibility

Pretreatment assessment should identify risks specific to each regimen: neuropathy or poorly controlled diabetes with EV, previous interstitial lung disease (ILD) or pneumonitis with deruxtecan-containing ADCs, and limited marrow reserve with SG. Active autoimmune disease requiring immunosuppression warrants careful assessment before checkpoint blockade [26,41]. Performance status, organ function, concurrent medication, and ability to attend monitoring are also relevant. Age alone should not determine suitability, but frail and substantially comorbid patients are less well represented in pivotal trials.

Shared decisions should address expected benefit, treatment schedule, monitoring, and symptoms that may impair daily life. Local approval and disease-specific recommendations must be checked for the exact regimen. For patients outside the studied population, particularly those with established ICI resistance or major comorbidity, an evidence-based alternative or a suitable trial is preferable to unvalidated use of the combination.

## 6. Maintaining Benefit During and After Treatment

### 6.1. Dosing, Scheduling, and Adverse-Event Management

Combination doses require evaluation rather than automatic adoption of monotherapy doses. In EVOKE-02, myelosuppression prompted reduction in the SG dose from 10 to 7.5 mg/kg in cohorts receiving pembrolizumab and carboplatin [52]. Serious toxicity in DESTINY-Lung03 did not support the evaluated T-DXd–durvalumab–platinum regimen in pretreated HER2-overexpressing NSCLC [53]. These findings justify dose-finding studies for new combinations; they do not support routine reduction in starting doses in established doublets.

Dose studies should consider efficacy, patient-reported symptoms, cumulative toxicity, and discontinuation alongside pharmacokinetics, rather than rely solely on early dose-limiting events [54]. Exploratory EV analyses suggest that benefit can persist after dose modification [55]. This supports toxicity-directed adjustment but does not establish equivalence of a lower starting dose. Patients treated for longer also have more opportunity to respond and require dose reduction.

Prompt management of regimen-specific toxicity helps patients continue treatment (Table 3). New pulmonary symptoms or infiltrates during T-DXd–ICI therapy require assessment for drug-related ILD/pneumonitis, infection, and other causes. Suspected ILD warrants interruption of T-DXd; confirmed grade ≥ 2 ILD requires permanent discontinuation and prompt systemic corticosteroids [56]. The ICI should be managed separately according to severity and the likelihood of immune-mediated injury. Fatal pulmonary events in T-DXd–ICI studies underscore the importance of early evaluation [57,58].

EV requires monitoring for skin reactions, neuropathy, and hyperglycemia. SG requires attention to neutropenia and diarrhea, with prophylactic granulocyte colony-stimulating factor for patients at increased risk of febrile neutropenia [26,59,60]. Diarrhea also requires assessment for infection and immune-mediated colitis. Dato-DXd warrants oral and ocular monitoring, while tisotumab vedotin requires label- or protocol-specified eye care [61,62]. ADC dose reduction, ICI interruption, and permanent discontinuation should follow drug-specific guidance. ICIs are not dose-reduced for toxicity [41].

A toxicity occurring during combination treatment cannot automatically be assigned to target expression in normal tissue. EV-related skin injury may involve Nectin-4 in the skin, whereas neuropathy is consistent with the MMAE payload; pneumonitis can arise during either drug class and is not a specific marker of on-target injury [26,41]. Table 3 distinguishes these possibilities. Real-world EV data offer useful context but not a clean estimate of the toxicity added by pembrolizumab: one cohort compared 132 first-line combination recipients with 157 later-line monotherapy recipients who differed in baseline fitness [63]. FAERS analyses identified stronger reporting signals for immune and respiratory events with the combination, but spontaneous reports lack a treated-population denominator and cannot provide incidence ratios [64].

**Table 3:** Selected safety findings in ADC–ICI studies.

Study	Regimen	Safety Population	Selected Findings	Mechanistic Assessment and Single-Agent Context
**A. Selected findings in randomized trials**				
EV-302 [26]	EV + pembrolizumab	Untreated la/mUC (440)	Rash (grouped term) 68% (grade 3–4, 15%); peripheral neuropathy 67% (grade 3–4, 8%); pneumonitis/ILD 10% (grade 3–4, 3.6%); ocular disorders 29% (grade 3–4, 0); increased glucose (laboratory) 66% (grade 3–4, 14%). EV dose reduction due to adverse reactions: 42%.	Skin injury: plausible Nectin-4-related on-target/off-tumor effect, with possible immune-mediated injury. Neuropathy: MMAE-related; ILD and hyperglycemia are not specific for target binding. Real-world comparisons are confounded by line and baseline fitness [63]. FAERS pneumonitis reporting OR 2.75 for combination vs. EV is a reporting signal, not an incidence ratio [64].
**RC48-C016** [9]	DV + toripalimab	Untreated HER2-expressing la/mUC (243)	Most frequent grade ≥ 3 TRAEs: increased γ-glutamyltransferase 7.4%, decreased neutrophil count 5.8%, hypokalemia 5.8%; serious TRAEs 28.4% vs. 40.5% with chemotherapy	Neutropenia is consistent with MMAE-mediated marrow injury. Liver enzyme changes and pneumonitis may have multiple causes; HER2-mediated normal-tissue injury is not established. No matched ADC-only comparator in this trial.
ASCENT-04 [59]	SG + pembrolizumab	Untreated PD-L1 CPS ≥ 10 TNBC (221)	Decreased neutrophil count (laboratory) 86% (grade 3–4, 50%); diarrhea 72% (grade 3–4, 12%); serious febrile neutropenia 7%; SG interruption 75%, dose reduction 35%, permanent discontinuation 7% (adverse-reaction-related)	Neutropenia and diarrhea are consistent with SN-38 toxicity, not demonstrated TROP2 on-target injury. Immune-mediated colitis remains a differential diagnosis. The comparator also contained pembrolizumab; it does not estimate toxicity added to SG by an ICI.
**OptiTROP-Lung05** [11]	sac-TMT + pembrolizumab	Untreated PD-L1 TPS ≥ 1% NSCLC; combination 208, pembrolizumab alone 204	Grade ≥ 3 TEAEs 55% vs. 31%.	Stomatitis and cytopenias are compatible with payload toxicity; TROP2-specific normal-tissue injury is not established. Pembrolizumab-only control estimates the added burden of the ADC, not the ICI.
OptiTROP-Lung06 [32]	sac-TMT + pembrolizumab vs. chemotherapy + pembrolizumab	Untreated PD-L1 TPS < 1% nonsquamous NSCLC; 455 randomized	Grade ≥ 3 treatment-emergent AEs 47.6% vs. 60.6%; stomatitis was predominantly grade 1–2.	Payload-associated mucosal injury is plausible; TROP2-mediated injury is unproven. Both groups received pembrolizumab, so this does not estimate the toxicity added by an ICI.
KATE2 [12]	T-DM1 + atezolizumab vs. T-DM1 + placebo	Previously treated HER2-positive advanced BC; 132/68	Serious AEs 33% vs. 19%; grade ≥ 3 thrombocytopenia 13% vs. 4%.	Thrombocytopenia is compatible with T-DM1 toxicity; HER2-dependent platelet injury is not established. This randomized ADC-fixed comparison shows an observed difference in serious AEs, but does not establish a mechanism or a real-world incidence estimate.
**B. Selected adverse events in early-phase studies**				
DS8201-A-U105 [57]	T-DXd 5.4 mg/kg + nivolumab	Pretreated HER2-positive MBC (29), HER2-low MBC (16), HER2-high mUC (30)	Adjudicated drug-related ILD/pneumonitis 20.7%, 0%, and 20.0%; one grade 5 event each in the HER2-positive MBC and mUC cohorts; median time to onset 193.0 and 135.5 days, respectively	ILD is a known T-DXd risk and can also be immune-mediated; HER2 on-target causation is not established. No randomized T-DXd-only comparison; rates across different tumor cohorts cannot quantify ICI-related excess.
BEGONIA arm 6 [58]	T-DXd + durvalumab	First-line hormone receptor-negative, HER2-low MBC (58)	Adjudicated drug-related ILD/pneumonitis 20.7% (grade 1–2, 19.0%; grade 5, 1.7%, reported by the investigator as COVID-19-related pneumonitis); pneumonitis was the most common AE leading to discontinuation of T-DXd (10.3%)	ILD can occur with T-DXd alone or ICI therapy; it is not proven HER2-mediated injury. No matched monotherapy control. The fatal event had an additional reported infectious context.
TROPION-Lung02 [33]	Dato-DXd + pembrolizumab without/with platinum chemotherapy	All treated: doublet 70, triplet 72; treatment-naive subsets: 42/54	Any-grade oral mucositis/stomatitis in treatment-naive patients: 61.9%/40.7%, mainly grade 1–2. Grade ≥ 3 TRAEs in all treated patients: 37.1%/59.7%; no treatment-related deaths.	Stomatitis is compatible with ADC/payload toxicity, but TROP2-mediated causation is not established. Platinum contributes to marrow toxicity. Doublet/triplet cohorts were not randomized and do not isolate any drug’s toxicity.
DESTINY-Lung03, part 1 [53]	T-DXd + durvalumab + cisplatin (arm 1A) or carboplatin (arm 1B); T-DXd alone (arm 1D)	Pretreated HER2-overexpressing NSCLC (11, 24, and 36)	Dose-limiting toxicities included grade 5 febrile neutropenia and grade 5 decreased platelet count (arm 1A); drug-related serious AEs 63.6%, 37.5%, and 16.7%; adjudicated drug-related ILD/pneumonitis 0, 12.5% (two grade 5 events), and 5.6% (grade 2 only)	Marrow toxicity can arise from T-DXd and platinum; ILD may involve T-DXd or durvalumab. No evidence establishes on-target HER2 injury. The monotherapy cohort provides context only because cohorts were nonrandomized and regimens differed in more than ICI use.
EV-103 cohort K [28]	EV + pembrolizumab	Cisplatin-ineligible la/mUC (76)	Grade ≥ 3 treatment-emergent AEs of special interest for pembrolizumab: severe skin reactions 19.7%, pneumonitis 5.3%. Treatment-related deaths: respiratory failure, pneumonitis, and sepsis (one each).	Skin injury may involve Nectin-4 and immune mechanisms; pneumonitis is not target-specific. The randomized EV-only arm is more informative than historical controls, but small numbers and different treatment durations limit estimation of rare-event risk [28].
RC48-C014 [27]	DV + toripalimab	la/mUC (41)	Any-grade TRAEs: increased AST 65.9%, increased ALT 63.4%, peripheral sensory neuropathy 63.4%; one treatment-related death from pneumonitis	Neuropathy is consistent with MMAE toxicity; transaminase elevations and pneumonitis have several possible causes. The study does not establish HER2-mediated toxicity or quantify added ICI risk.
Matulonis et al. [38]	Mirvetuximab soravtansine + pembrolizumab	Platinum-resistant ovarian cancer (56)	Treatment-emergent pneumonitis 25% (grade ≥ 3, 4%); diarrhea 57% (grade 3, 4%)	Payload toxicity, immune pneumonitis/colitis, and other causes require clinical assessment. FRα-mediated causation is not established. No concurrent mirvetuximab-only group; historical monotherapy rates cannot quantify the excess risk.

Percentages refer to the indicated treated population unless a subgroup is specified. Serious AEs meet regulatory seriousness criteria, such as hospitalization or death; grade ≥ 3 AEs describe severity and are not synonymous with serious AEs. Laboratory abnormalities, clinical AEs, TRAEs, and TEAEs are distinct categories. The mechanistic column gives plausible explanations, not adjudicated causes. On-target/off-tumor injury means ADC binding to its target in normal tissue; payload-mediated or immune-mediated toxicity is not automatically on-target. Cross-study rates differ in population, treatment line, exposure, and ascertainment and cannot estimate the toxicity added by an ICI. FAERS reporting odds are not incidence rates or relative risks. Dose modifications apply to the named drug. ALT, alanine aminotransferase; AST, aspartate aminotransferase; ILD, interstitial lung disease; other abbreviations as in Table 1.

### 6.2. Treatment Duration and Decisions After Response

Protocol-specified duration, actual time on treatment, and a clinically justified stopping decision are different. Table 4 consolidates reported component-specific treatment durations from three positive phase III trials. In EV-302, EV could continue until progression or unacceptable toxicity, whereas pembrolizumab was limited to 35 three-week cycles. Median time receiving any study treatment was 9.4 months versus 4.1 months with chemotherapy, which was limited to six cycles [26]. Regimen-level duration includes periods when only one drug was continued.

RC48-C016 imposed no fixed maximum number of cycles for either drug and permitted one to continue after the other was discontinued. Median treatment delivery was 14 cycles for each drug, and 91 of 243 patients (37.4%) remained on treatment at cutoff [9]. The component-specific medians in Table 4 therefore do not define a recommended course of approximately eight months.

In ASCENT-04, treatment duration differed between the ADC–ICI and chemotherapy–ICI groups (Table 4) [10]. US prescribing information specifies SG until progression or unacceptable toxicity and pembrolizumab for up to approximately 24 months. Adverse reactions led to SG interruption in 75%, dose reduction in 35%, and permanent discontinuation in 7% [59]. Continuing treatment frequently required adjustment rather than uninterrupted delivery at the starting dose.

Values describe the cited analysis, not optimal treatment duration. Cycles, weeks, and months retain the original reporting units. Protocol caps differ from observed medians. Medians for different drugs or endpoints cannot be subtracted to estimate a treatment-free interval. This is a selected summary of trials with reported component-specific medians, not an exhaustive inventory. Abbreviations are defined in Table 1.

These observations support regular reassessment and protocol- or label-directed dose modification, not elective cessation at the reported median treatment time. Actual duration is influenced by progression, toxicity, protocol limits, and incomplete follow-up. PFS and treatment duration also start and end according to different definitions; subtracting their medians does not estimate a treatment-free interval.

After a response, review disease control, cumulative symptoms, organ function, and patient preference at each treatment assessment. Continue the regimen within its protocol or prescribing limits while clinical benefit outweighs treatment-related risks. If toxicity develops, interrupt, reduce, or discontinue the ADC suspected of causing the adverse event as specified for that drug; manage the ICI separately, without dose reduction. Continuing one drug after the other must stop may be permitted for a particular regimen, but this should not be conflated with a planned ICI-maintenance strategy. Complete radiological response alone is not an established reason to stop both drugs.

To establish a less intensive approach, trials should randomize patients with controlled disease to continued combination treatment, ADC withdrawal, or a reduced ADC schedule. Outcomes should be measured from that decision and include PFS, symptoms, quality of life, and results of retreatment. Response depth and ctDNA may help define research subgroups, but neither currently supplies a validated stopping rule across ADC–ICI regimens.

### 6.3. Resistance and Treatment After Progression

Resistance mechanisms may inform subsequent treatment. Reduced target expression, impaired internalization or intracellular processing, drug efflux, and payload resistance can all limit ADC efficacy [1]. Samples obtained after T-DXd progression have shown HER2 loss and ERBB2 alterations affecting antibody binding [2]. TOP1 mutations can reduce sensitivity to both SN-38 and DXd, potentially causing cross-resistance between ADCs with different targets but related payloads [3]. Clinical evidence that checkpoint blockade prevents these changes is lacking.

In the retrospective ADC-Low study, median PFS with the second of sequential SG and T-DXd treatments was 2.7 months, and 54.4% of patients had primary resistance [65]. Later treatment line and adverse disease characteristics may partly explain these outcomes; they do not establish universal cross-resistance. Switching payload class restored activity in selected preclinical models, but clinical validation is needed [66].

After progression, treatment should follow disease-specific evidence, considering previous benefit, residual toxicity, organ function, and current target status when it can change management. Repeat biopsy may identify actionable changes or support trial enrollment. HER2 loss, payload resistance, and impaired antigen presentation suggest different approaches, but no validated treatment algorithm follows from these findings alone [2,3,21,23]. Continuing the same ICI with a different ADC after progression requires direct clinical evidence.

### 6.4. Priorities for Clinical Research

Ongoing trials address several unresolved choices. TroFuse-011 compares sac-TMT alone, sac-TMT plus pembrolizumab, and chemotherapy in advanced TNBC with CPS < 10; TROPION-Breast05 includes a Dato-DXd monotherapy group in selected countries [67,68]. These designs can assess whether adding an ICI improves outcomes over the ADC alone. TROPION-Lung07 and DESTINY-Lung06 evaluate first-line lung cancer regimens; SGNDV-001 and DESTINY-Gastric05 address urothelial and gastric cancer [69,70,71,72]. TROPION-Lung10 also evaluates an additional immune target [73]. Results are needed before these approaches guide treatment.

Sequencing trials should compare upfront combination therapy with a planned sequence and document how many patients actually receive the subsequent treatment. PFS2, measured from initial randomization to progression on the next treatment or death, can complement OS, cumulative toxicity, and quality-of-life assessment. Duration trials should also distinguish planned withdrawal from treatment changes required by toxicity.

Translational studies should evaluate biomarkers that could change treatment decisions. Serial tissue and blood samples may help select patients for duration trials or identify options after progression, but biomarker-guided management requires prospective validation. Studies including older and comorbid patients should also assess early discontinuation, hospitalization, and functional outcomes to determine whether trial benefits are maintained in routine care.

## 7. Integrating Radiotherapy with ADC–ICI Therapy

### 7.1. Biological Rationale and Payload-Specific Effects

Radiotherapy has two distinct rationales in this setting: eliminating a clinically important local lesion and modifying antitumor immunity. Radiation-induced cytosolic DNA can activate cyclic GMP–AMP synthase (cGAS)–STING and type I interferon signaling, whereas TREX1 can attenuate this response [74]. Radiation may also damage circulating lymphocytes and normal tissue. Its interaction with an ADC therefore depends on the payload, irradiated volume, dose, fractionation, and timing; greater local cytotoxicity is not equivalent to systemic immune synergy.

Targeted monomethyl auristatin E (MMAE) conjugates combined with radiation produced CD8-dependent tumor control and enhanced checkpoint blockade in mouse models [75]. Subsequent experiments comparing MMAE- and DXd-containing conjugates found payload-dependent differences in radiosensitization and the effect of PD-1 blockade [76]. These findings support studying a defined ADC–radiotherapy–ICI regimen rather than assuming a class effect. Demonstrating an immune contribution requires functional immune experiments; demonstrating synergy additionally requires a prespecified comparison against the expected combined effects of the individual treatments.

### 7.2. Direct and Indirect Clinical Evidence

Direct clinical evidence for ADC–ICI therapy with radiotherapy remains limited. A case report described regression and symptomatic improvement after radiotherapy with EV and pembrolizumab for recurrent metastatic urothelial carcinoma [77]. Following maximal transurethral resection, six patients with HER2-expressing, nonmetastatic muscle-invasive bladder cancer received DV, toripalimab, and radiotherapy; five achieved a complete response and two experienced grade 3 treatment-related adverse events [78]. These observations support feasibility in selected patients, but a local bladder-preservation outcome cannot establish prolonged control of metastatic disease. Neither report isolates the effect of radiotherapy or demonstrates pharmacological synergy.

ADC–radiotherapy studies provide indirect evidence only. In the prospective observational COMBART cohort, 40 patients with metastatic breast cancer received T-DXd and radiotherapy. Among 22 treated for oligoprogression, the median interval from radiotherapy to a change in systemic treatment was 19.1 months [79]. There was no ICI regimen or randomized comparator. This interval may reflect selection of patients with otherwise controlled disease and clinician decisions about switching therapy; it is not a comparative PFS or survival estimate.

Randomized ICI–radiotherapy studies provide both supportive and contrary evidence, without testing an ADC. A pooled analysis of two trials in 148 immunotherapy-naive patients with metastatic NSCLC reported more out-of-field responses and longer median PFS with pembrolizumab plus radiotherapy than with pembrolizumab alone (9.0 versus 4.4 months; HR 0.67, 95% CI 0.45–0.99) [80]. In contrast, CHEERS analyzed 96 patients with advanced solid tumors and found no significant PFS improvement from irradiation of up to three lesions during ICI treatment (4.4 versus 2.8 months; HR 0.95, 95% CI 0.58–1.53), despite 75% local control in irradiated patients [81]. These studies differ in histology, burden, and schedule. Their results illustrate why local control, out-of-field response, and overall PFS must be reported separately.

The mixed ICI–radiotherapy evidence argues against assuming that irradiation will improve any ADC–ICI regimen. It nevertheless identifies testable questions about out-of-field response, limited progression, and treatment continuation. In this review, radiotherapy is therefore considered a potential way to address specific patterns of treatment failure, not an established means of enhancing the systemic efficacy of ADC–ICI therapy.

### 7.3. Recurrent and Metastatic Settings for Prospective Evaluation

The intended role of radiotherapy should determine eligibility and endpoints. For symptomatic recurrence or metastases, the immediate objective is palliation or prevention of local complications; symptom relief does not establish an improvement in systemic PFS. An isolated locoregional recurrence potentially suitable for curative salvage requires a disease-specific multidisciplinary assessment and should not be grouped with widespread metastatic disease.

For oligoprogression, a reasonable research population comprises patients with a small number of progressing lesions and continued control of the remaining disease during a tolerable ADC–ICI regimen. Trials should compare radiotherapy plus continuation with the appropriate alternative: continued systemic therapy where reasonable, or a change in systemic treatment where progression otherwise warrants a switch. De novo oligometastatic disease, residual lesions after response, and oligoprogression are not interchangeable, and evidence from one setting should not be generalized to the others.

Such trials should measure PFS from the local-treatment decision, with progression outside the irradiated field, time to the next systemic treatment, symptoms, and quality of life as distinct outcomes. To investigate an abscopal effect, measurable nonirradiated lesions and prespecified response assessment are needed. Serial tissue or blood measurements may support a mechanism, but ctDNA decline or immune-cell changes alone would not prove that radiation improves systemic control.

### 7.4. Safety and Treatment Scheduling

ADC–radiotherapy safety data cannot establish the safety of adding an ICI. In a retrospective study of 98 patients, concurrent ADC treatment and stereotactic radiotherapy for brain metastases were associated with symptomatic radiation necrosis, with particular concern for larger or previously irradiated lesions [82]. COMBART also documented late pulmonary and central nervous system toxicities [79]. These findings warrant caution but do not define a uniform risk for every ADC, radiation schedule, or three-modality regimen.

Planning should account for previous radiation dose, target volume, organ-at-risk exposure, and current drug-related injury. Thoracic irradiation is especially relevant when the ADC and ICI can both cause pneumonitis; brain reirradiation requires assessment of cumulative dose and necrosis risk. Trials should specify drug timing and interruption rules and prospectively record lymphopenia, steroid use, radiation-field and out-of-field injury, late toxicity, and discontinuation of each drug. No universal drug-holding interval or preferred sequence is established. Outside trials, radiotherapy should have a defined local indication; routine addition solely to extend systemic disease control is not supported.

## 8. Conclusions and Future Directions

ADC–ICI therapy improves outcomes in defined advanced solid-tumor populations. In this review, prolonged disease control is supported principally by randomized PFS results, with DoR describing durability among responders. OS, symptoms, quality of life, and toxicity determine its broader clinical value. These findings establish the efficacy of specific regimens, not universal pharmacological synergy. Selection for recurrent or metastatic disease should follow the relevant trial population and account for prior perioperative therapy, treatment-free interval, and residual toxicity.

Observed treatment durations do not establish optimal stopping rules. Randomized studies should determine whether treatment can be shortened or modified while preserving control. For radiotherapy–immunotherapy research, the relevant next question is whether local treatment of selected residual or progressing lesions improves outcomes during otherwise effective ADC–ICI therapy. Direct three-modality evidence is not sufficient to recommend routine use. Future studies must distinguish local palliation, delayed systemic progression, and an immune-mediated effect outside the radiation field, while measuring late toxicity and patients’ ability to continue treatment.

## Figures and Tables

**Table 2:** Clinical implications of current evidence for ADC–ICI therapy.

Clinical Setting	Comparative Evidence	Main Uncertainty	Treatment Consideration
Untreated advanced urothelial carcinoma [8,9,35]	Phase III PFS and OS benefit over platinum chemotherapy; longer follow-up supports sustained benefit with EV–pembrolizumab.	No direct comparison of the two regimens; DV evidence is restricted to HER2 IHC 1+ to 3+.	Choose the exact regimen according to local approval and guidelines; match eligibility, HER2 requirements, and neuropathy or other toxicity risks.
Untreated PD-L1 CPS ≥10 advanced TNBC [10]	Phase III PFS benefit from SG–pembrolizumab over chemotherapy–pembrolizumab.	OS remains immature; no comparison with SG alone.	Use only where locally approved or recommended for the studied first-line population; evaluate marrow reserve and diarrhea risk.
Untreated PD-L1 TPS ≥1% advanced NSCLC without targetable alterations [11]	Phase III PFS benefit from sac-TMT–pembrolizumab over pembrolizumab alone.	Longer follow-up and comparison with chemoimmunotherapy are needed.	A positive trial does not by itself establish local standard of care. Check regulatory status and alternatives, including chemoimmunotherapy for eligible TPS 1–49% patients.
Previously treated HER2-positive or hormone receptor-positive/HER2-negative advanced breast cancer [12,13]	No significant PFS benefit in KATE2 or SACI-IO HR+.	Exploratory PD-L1 subgroup findings are unconfirmed.	Routine use in the overall populations studied is not supported.
Gastric and gynecologic cancers [36,37,38]	Early-phase response and survival estimates; no randomized evidence of benefit.	Comparative efficacy and survival benefit remain unestablished.	Prioritize trial enrollment; nonrandomized findings do not establish a replacement for standard treatment.
Untreated PD-L1 TPS < 1% nonsquamous advanced NSCLC [32]	Phase III PFS benefit with sac-TMT–pembrolizumab over platinum–pemetrexed–pembrolizumab.	Interim follow-up; OS immature; no sac-TMT-only group.	Apply only to the exact studied population and locally authorized indication; this does not validate all ADC–ICI regimens in PD-L1-negative disease.
Post-platinum advanced urothelial carcinoma [29,30]	No efficacy improvement in MORPHEUS-UC; a PFS signal in JAVELIN Bladder Medley maintenance.	Small cohorts; different treatment settings; JAVELIN survival analyses incorporated external controls.	Do not equate progression after platinum with nonprogressing maintenance populations; confirmation is required before routine SG–ICI use.
Pretreated HER2-positive MSS/pMMR metastatic colorectal cancer [39]	Limited single-arm activity with DV–pembrolizumab: ORR 13.3%, median PFS 2.9 months.	Only 15 patients; no randomized comparison.	HER2 expression alone does not justify extrapolation from urothelial cancer; use disease-specific options or a trial.

Evidence categories describe trial results, not regulatory approval. Treatment requires confirmation of the exact indication, local authorization, and disease-specific guidance. The table does not rank regimens across trials or imply pharmacological synergy. Early signals and negative results are retained where they affect a treatment decision; Table A1 provides complementary early-phase data. Abbreviations are defined in Table 1.

**Table 4:** Observed treatment duration in selected positive phase III ADC–ICI trials.

Trial and Analysis	Median Time on ADC	Median Time on ICI	Protocol Limits and Interpretation
EV-302 primary analysis [8,26]	EV 7.0 months	Pembrolizumab 8.5 months	EV until progression/unacceptable toxicity; pembrolizumab up to 35 cycles. Any study treatment: 9.4 months vs. 4.1 months for chemotherapy (maximum six cycles).
RC48-C016 interim analysis [9]	DV 34.1 weeks; 14 cycles	Toripalimab 35.0 weeks; 14 cycles	No fixed maximum cycle number; one drug could continue after the other stopped. 91/243 patients (37.4%) remained on treatment at cutoff.
ASCENT-04 primary PFS analysis [10,59]	SG 8.9 months	Pembrolizumab 8.5 months	Control-arm medians: chemotherapy 6.2 months; pembrolizumab 6.4 months. SG until progression/unacceptable toxicity; pembrolizumab capped at approximately 24 months.

## Data Availability

No new datasets were generated for this narrative review. The evidence discussed is available in the cited publications and regulatory documents.

## Appendix A. Early-Phase Studies in Urothelial, Breast, and Lung Cancers

The early-phase studies in Table A1 provide context for the randomized evidence and identify questions for further investigation. RC48-C014 provides the developmental context for RC48-C016; DS8201-A-U105 and BEGONIA arm 6 show that responses can coexist with pulmonary toxicity and an unmet response objective; BEGONIA arms 7/8, TROPION-Lung02, and EVOKE-02 inform response duration, PD-L1 selection, and subsequent trial design. Inclusion is independent of whether results are encouraging. These studies do not establish comparative efficacy, the benefit of adding an ICI, or pharmacological synergy.

**Table A1:** Early-phase ADC–ICI studies informing response durability, patient selection, and safety.

Study and Design	Tumor Type and Setting	Regimen	n	Confirmed ORR, % (95% CI)	Median DoR, mo (95% CI)	Median PFS, mo (95% CI)	Median OS, mo (95% CI)	High-Grade AEs, % (Definition Specified)
**A. Urothelial carcinoma**								
RC48-C014 [27] Phase Ib/II	la/mUC; 61% untreated; HER2-unselected	DV 1.5–2.0 mg/kg during escalation; RP2D 2.0 mg/kg + toripalimab 3 mg/kg q2w	41	73.2 (57.1–85.8)	8.6 (7.2–16.8)	9.3 (5.7–11.2)	33.1 (16.5–NE)	Grade ≥ 3 TRAEs 51.2
DS8201-A-U105 [57] Phase Ib	Pretreated HER2-high mUC (cohort 3)	T-DXd 5.4 mg/kg + nivolumab 360 mg q3w	30	36.7 (19.9–56.1)	13.1 (4.1–NE)	6.9 (2.7–14.4)	11.0 (7.2–NE)	Grade ≥ 3 TEAEs 70.0
**B. Breast cancer**								
DS8201-A-U105 [57] Phase Ib	Pretreated HER2-positive MBC (cohort 1)	T-DXd 5.4 mg/kg + nivolumab 360 mg q3w	32	65.6 (46.8–81.4)	NE (7.9–NE)	11.6 (6.9–NE)	NE (20.8–NE)	Grade ≥ 3 TEAEs 51.7 ^a^
DS8201-A-U105 [57] Phase Ib	Pretreated HER2-low MBC (cohort 2)	T-DXd 5.4 mg/kg + nivolumab 360 mg q3w	16	50.0 (24.7–75.3)	5.5 (2.8–8.0)	7.0 (2.3–10.8)	19.5 (2.7–NE)	Grade ≥ 3 TEAEs 43.8
BEGONIA arm 6 [58] Phase Ib/II platform	First-line hormone receptor-negative MBC; locally assessed HER2-low	T-DXd 5.4 mg/kg + durvalumab 1120 mg q3w	58	62.1 (48.4–74.5)	15.2 (8.4–NC)	12.6 (8.4–16.3)	30.3 (18.8–NC); immature	Grade 3–4 AEs 48.3
BEGONIA arms 7/8 [31] Phase Ib/II platform	First-line TNBC; arm 7 PD-L1-unselected/arm 8 PD-L1-high	Dato-DXd 6 mg/kg + durvalumab 1120 mg q3w	62/33	79.0 (66.8–88.3)/81.8 (64.5–93.0)	17.6 (10.5–27.3)/immature	14.0 (11.0–21.1)/immature	NR/—	Grade 3–4 AEs 59.7/36.4
**C. Non-small-cell lung cancer**								
TROPION-Lung02 [33] Phase Ib	First-line NSCLC without actionable genomic alterations	Dato-DXd 4 or 6 mg/kg + pembrolizumab 200 mg q3w, without/with platinum chemotherapy	42/54 ^b^	54.8/55.6	20.1/13.7	11.2/6.8	—	Grade ≥ 3 TRAEs 37.1/59.7 Safety n = 70/72 ^b^
EVOKE-02 cohorts A/B [34] Phase II	First-line NSCLC without actionable alterations; PD-L1 TPS ≥ 50%/<50%	SG 10 mg/kg days 1 and 8 + pembrolizumab 200 mg q3w	30/62	66.7 (47.2–82.7)/29.0 (18.2–41.9)	—	13.1 (6.7–NR)/7.0 (4.2–12.9)	—	Grade ≥ 3 TEAEs 76.1 (pooled n = 92)

CIs are 95%; —, not tabulated. RC48-C014 includes dose escalation (n = 6) and expansion (n = 35). ^a^ DS8201-A-U105 cohort 1 efficacy includes three dose-escalation patients (n = 32); its displayed safety result refers to part 2 at 5.4 mg/kg (n = 29). HER2-positive breast cancer was IHC 3+ or IHC 2+/ISH-positive; HER2-low was IHC 1+ or IHC 2+/ISH-negative; HER2-high UC was IHC 2+ or 3+. BEGONIA arm 6 used local HER2 assessment and did not meet its prespecified response objective. In BEGONIA arm 8, PD-L1-high was defined by a tumor area positivity score ≥ 10% with the SP263 assay. ^b^ TROPION-Lung02 efficacy refers to untreated patients (42/54); safety refers to all treated patients (70/72). Cohorts were not randomized against each other; biomarker thresholds and AE definitions are study-specific. Dato-DXd, datopotamab deruxtecan; mUC, metastatic urothelial carcinoma; NC, not calculable; q2w/q3w, every 2/3 weeks; RP2D, recommended phase II dose; other abbreviations as in Table 1.
